# Where extended reality and AI may take us: Ethical issues of impersonation and AI fakes in social virtual reality

**DOI:** 10.1371/journal.pone.0340829

**Published:** 2026-04-02

**Authors:** Ramon Oliva, Michael Wiesing, Jaime Gallego, Masahiko Inami, Victoria Interrante, Anatole Lécuyer, Rachel McDonnell, Florian Nouviale, Xueni Pan, Frank Steinicke, Mel Slater

**Affiliations:** 1 Event Lab, Department of Clinical Psychology and Psychobiology, University of Barcelona, Barcelona, Spain; 2 Research Center for Advanced Science and Technology, University of Tokyo, Tokyo, Japan; 3 Department of Computer Science and Engineering, University of Minnesota, Minneapolis, United States of America; 4 Hybrid Research Team, Inria Rennes/IRISA, Rennes, France; 5 School of Computer Science and Statistics, Trinity College Dublin, Dublin, Ireland; 6 Department of Computing, Goldsmiths University of London, London, United Kingdom; 7 Department of Informatics, Universität Hamburg, Hamburg, Germany; 8 Institute of Neurosciences of the University of Barcelona, Barcelona, Spain; University of Antwerp, BELGIUM

## Abstract

We describe a study based on a panel discussion that took place in social virtual reality (VR) at the conference IEEE VR 2025. Each panellist was embodied in a virtual body that looked like themselves. The VR scene was projected onto a screen in front of the audience in the main conference theatre. During the course of the panel two of the panellists swapped avatars, but tried to act as if they were the other person. Additionally, a large language model-controlled Alan Turing (AT) avatar participated. The study aimed to assess the audience’s ability to detect the human identity swap and their perception of the AT panellist. We found that about 40 of 100 attendees who answered a post panel survey did not notice the body swap, highlighting a form of change blindness towards social identity in VR. While AT was seen as less realistic and somewhat distracting, its inclusion demonstrated the increasing capabilities of AI in natural language processing and interaction. The paper emphasises the critical need for ethical considerations, such as identity verification and guidelines for representing historical figures, as virtual reality platforms that can represent historical figures in combination with LLMs become more widespread.

## Introduction

Over the last decade virtual reality (VR) has emerged out of university and industrial laboratories to be on its way to becoming a consumer product. Global companies have envisaged a ‘metaverse’ where millions can be simultaneously present in a shared virtual environment and interact together in almost all activities of their lives. This vision would go beyond VR to encompass augmented and mixed reality (extended reality, XR), where the totality of what we call ‘reality’ would become a blend of the virtual and physical, which would be normal for future generations. While for XR this is still a (likely) possibility, in the domain of artificial intelligence (AI), and especially in natural language processing based on large language models (LLM), the change in just the past three years has been enormous, with billions of people interacting with LLMs in their everyday lives, as a matter of course. Just a few years ago, the possibility of conversing with a computer program as if it were a real person seemed far off in time. Indeed, for decades no computer program was capable of passing the Turing Test [1], and as recently as 2024 it was still noted that no system had successfully done so [2] (“Now, the Turing test has not yet been passed by any AI system”, p26). Today, with tools like ChatGPT and other LLMs, natural, language-based interaction with computers has become a part of everyday life for many. However, as these technologies mature and become more accessible, ethical concerns surrounding identity and impersonation in XR [3], as well as the possibility of AI systems masquerading as real people, have resulted in growing concern [4]. While there is a lot of speculation about possible ethical dangers of VR, there is little empirical evidence [5].

In the AI realm there is a great deal of discussion about the dangers for humanity – including predictions about the end of democracy or even the end of human civilization [6]. It is recognized, however, that both AI and XR can have profound positive impacts in society – for example, in the health care sector for efficiently identifying tumours [7]. In VR the positive uses have been highlighted, such as reducing racial bias via body ownership illusions [8–11] or enhancing prosocial behaviour [12]. There have been multiple examples of its use for medical education – e.g., the meta-studies of [13,14] – and for pain relief [15]. It has also been used in psychological therapy since the 1990s [16].

VR creates four key illusions: (i) Place Illusion (PI), (ii) Plausibility (Psi), (iii) body ownership, and (iv) co-presence, [17]. PI and Psi together constitute ‘presence’. PI refers to the illusion of being in the space depicted by the VR, based on sensorimotor contingencies—that is, perceiving the virtual world by the use of the body similarly to how we operate in reality [18]. Psi is the illusion that virtual events are actually occurring, enhanced when the environment responds naturally to the presence and actions of participants, such as a virtual crowd parting to let the participant move through, or a virtual character making brief eye contact.

Body ownership occurs when participants perceive a life-sized virtual body from a first-person perspective that substitutes their real body and synchronizes visually, tactually, and in its movements with their own body [19]. Changes in this virtual body can influence perception and behaviour—for example, embodying a Black virtual body reduces implicit racial bias in White participants [11]. Co-presence is the sense of sharing space with others in VR, building upon the previous illusions. It facilitates realistic social interactions among participants, as demonstrated, for example, by [20].

These illusions, while integral to VR’s ability to simulate realistic environments, raise important questions about how people might be deceived into sharing sensitive information with malicious actors who exploit them to masquerade as a trusted friend, colleague, or family member, by adopting their virtual body. This includes not only conventional data breaches or impersonation, but also the possibility of AI systems or malicious users assuming the appearance and behavioural likeness of real individuals. Studies have shown that AI chatbots can convincingly impersonate real people on traditional social media. For example, Radivojevic, Clark, et al. [4] found that users correctly identified AI-operated accounts only 42% of the time during online political discussions.

In immersive virtual environments, this impersonation capability is particularly concerning. Modern off-the-shelf VR systems not only deliver audio-visual and some haptic stimuli but are also equipped with increasingly sophisticated sensors to precisely track the body movements of participants, translating these into avatar movements and can also include tracking sensors for eye movements or facial expressions. Consequently, VR systems effectively function as sensitive biometric measurement devices [21]. Previous research has shown that head and hand movement data can be used to identify people [22,23] or infer their login information [24]. Thus, malicious actors could potentially record a user’s distinct movements, gestures, and voice, subsequently training an AI to replicate these biometric signatures. Such trained AI could then convincingly control an avatar closely resembling the original participant, dramatically increasing risks associated with identity theft, unauthorized impersonation, and breaches of personal trust in immersive social contexts. It has been estimated that more than 5 billion people in the world use social media [25], with more than 240 million new users each year, posting photos and videos of themselves and family and friends, thus providing a great amount of information useful for constructing their avatar without their knowledge.

Here we concentrate on two themes mentioned above. The first is false identity: is it possible for people to embody a virtual body that looks like someone else, without others present actually noticing? If so, a person might divulge sensitive information, thinking that they were interacting with friends or family. Second, we consider ethical problems surrounding using LLMs in the context of VR to simulate a real human – in this case of the famous historical figure Dr Alan Turing.

In order to investigate these issues, we set up a panel discussion at the international conference IEEE VR 2025, with the title ‘Where will extended reality and AI take us?’ [26]. During this event, the panellists participated in a session that began in a virtual environment using Quest 3 head-mounted displays (HMDs), embodying avatars closely resembling their real selves with synchronized head and hand tracking. Crucially, after 15 minutes, a deliberate body swap occurred between two panellists: Frank Steinicke (FS) and Mel Slater (MS). Following the swap, each of these consciously attempted to adopt the identity, mannerisms, and conversational style of the other, reinforced by the moderator Xueni Sylvia Pan who addressed their corresponding avatars by the names associated with their appearance, even though she knew that they were swapped.

A virtual human character with appearance modelled on Dr Alan Turing was part of the panel, speaking via an interface to ChatGPT4o. Throughout this paper we use ‘AT’ to refer to this avatar, and ‘Alan Turing’ to refer to the real person. This scenario aimed to assess two main issues: first, whether viewers would notice the deliberate avatar swap, or whether they would exhibit a type of ‘change blindness’, and not notice the swap between the two panellists. Second, we were interested in how the audience would perceive the AI panellist and its contribution to the panel discussion.

## Materials and methods

### Ethics

**Fig 1:**
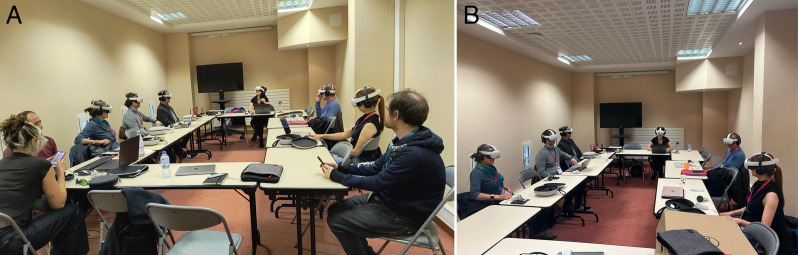
The panellists were in a private room away from the main conference, each wearing a head-mounted display through which they could see and interact with virtual representations of one another. **(A)** During rehearsals with others present **(B)** During the actual panel.

**Fig 2:**
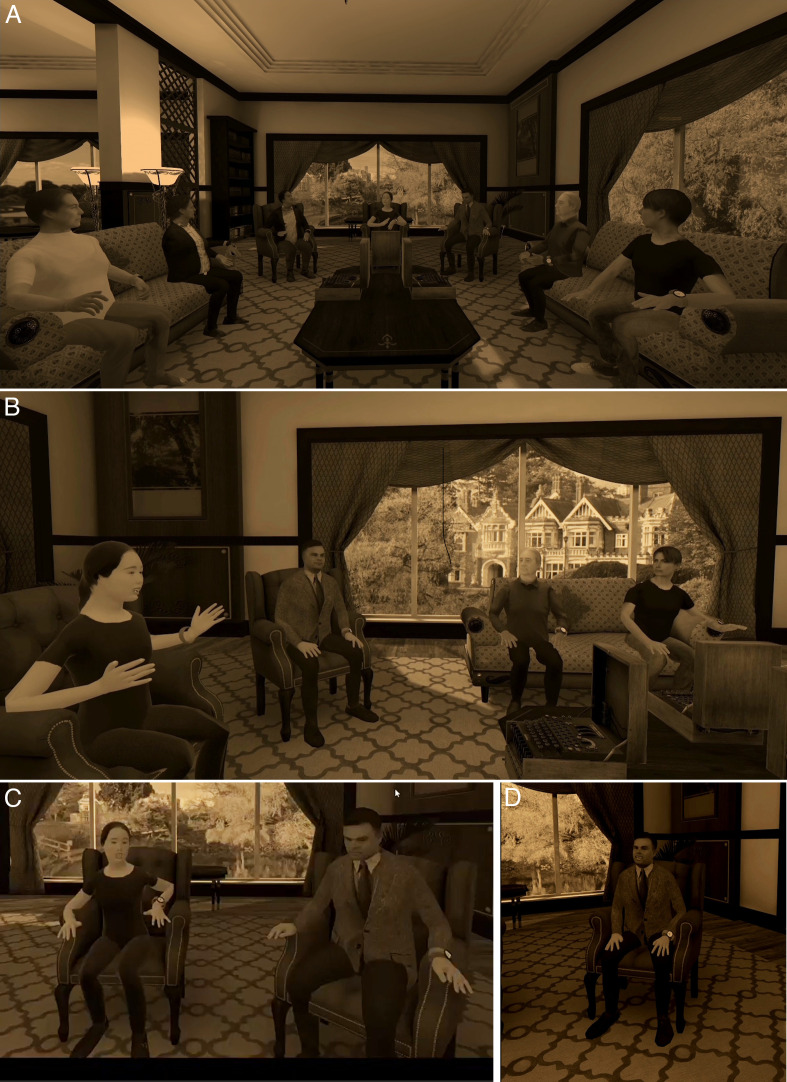
The virtual room where the discussion took place. **(A)** An overview of the room showing all the panellists. **(B)** The Moderator is speaking, and note also the view through the window. **(C)** The spatial relationship between the Moderator and AT. **(D)** The AT avatar speaking.

**Fig 3:**
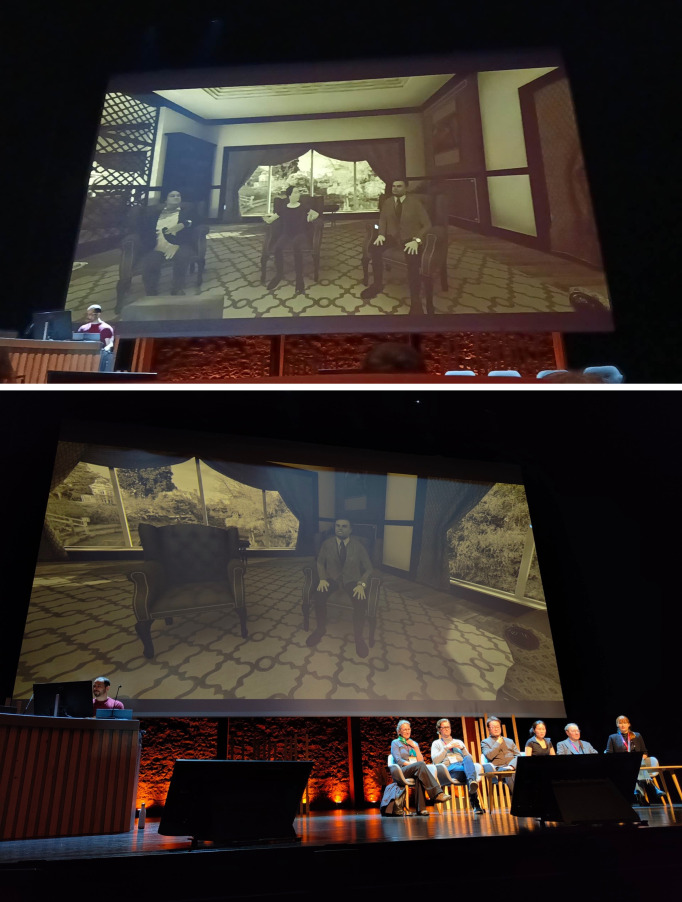
Views from the audience. Top: an audience view showing also RO operating and recording the video. Bottom: By the end of the virtual panel all the panellists were on stage while AT continued to be displayed.

The data for this study were obtained by a survey of the audience who attended the panel discussion. Audience members were shown a QR code projected on the theatre screen, and they could use the camera on their mobile phones to access the questionnaire, and were also free not to do this. No identifying data was recorded about participants. The Comissió Bioètica of the University of Barcelona were consulted and decided that ethical approval was not needed for this study on the grounds that no demographic or identifying data were collected, and the activity was therefore considered a collection of panel feedback rather than a formal research study. The individuals portrayed in Figs 1-3 are the authors who were panellists, or their representations depicted in virtual reality, and have given written informed consent to publish these images.

### Setup of the panel

The panel members were physically together in a private room in the conference centre, out of bounds to other attendees (Fig 1). Each wore a Quest 3 HMD on which there was a pre-installed version of a program called ‘VR United’ [27]. Additionally, they could hold the controllers for hand tracking or put the controllers down and directly use the Quest hand tracking as they preferred. VR United displayed a room modelled on one in Bletchley Park, the place where Dr Alan Turing led the work on codebreaking during the Second World War.

In order to create this room an asset store package was taken as the base [28] and some changes were made to the distribution of the room. Further details were added to transport the participants to the WWII period, such as a portrait of the King George VI [29], a picture of Prime Minister Winston Churchill [30], and a map showing the evolution of the war [31]. Also several Enigma code-breaking machines were placed on the table in front of the participants [32]. For the background, photographs from Bletchley Park were used [33], so what could be seen outside through the windows corresponds to reality (at least, how it looks today). Additionally, a full screen shader was created in order to apply a sepia tone to give an impression of being from the past.

Each of the panellists had previously submitted a single photograph of themselves standing upright facing the camera which was used to create their look-alike avatar. VR United supports multiple people being simultaneously present in the same virtual space, each with their own look-alike virtual body, in this case in a room in the virtual Bletchley Park model. They were all seated and could see each other’s avatar when looking around based on the Quest 3 head tracking (Fig 2).

The panellists rehearsed several times over several days, mainly concentrating on technical issues. For example, normally VR United is used by people who are in different physical locations, so that they hear each other only through the Quest auditory output. In this case since the panel members were in the same room, each utterance would be heard twice, first the direct one when a person spoke, followed by the Quest output with a short delay, due to network latency. Therefore, the panel members turned off the Quest sound, except when AT spoke. In these rehearsals the panel members agreed on the questions that would be discussed during the panel.

On the stage in the auditorium where the panel took place there were chairs set out for the panellists. One of the authors (AL) was on stage introducing the panel and pretending that he was waiting for the panellists to appear. At the same time another of the authors (RO) was on stage working on a PC. He started up the VR United application and initiated the virtual Bletchley Park room. RO acted as the operator throughout the panel, invisibly in the virtual room, and also making a video of the discussion from within the virtual environment. Once RO started the application, and all the panellists had joined it, the large auditorium screen displayed it.

The moderator of the panel (one of the authors, XP) had the virtual AT sitting to her left, and the panellists seated around in a circle. She started the panel discussion by asking the members to introduce themselves and then turned to the AT character and asked ‘him’ to introduce himself, which was accomplished via an interface to ChatGPT. Then the panel discussion started, led by XP, and every so often she would ask for comments from AT. See https://youtu.be/fl0vJ5-FlsQ for a video overview.

After 15 minutes the operator RO triggered the body swap so that MS and FS were embodied in each other’s virtual bodies and tried to imitate the voice and behaviours and even comments that they would expect the other to make. For example, MS in the virtual body of FS (from Hamburg) referred to work happening in Hamburg, and FS in the virtual body of MS (from Barcelona) similarly referred to work in Barcelona. The moderator XP and the other panellists addressed their corresponding avatars by the names associated with their appearance, except in one instance when XP mistakenly looked at the FS body and referred to ‘Mel’, but this was quickly corrected.

During the next 15 minutes, one by one the panellists quit from VR United, and the audience would see their avatars vanish. The panellists quickly made their way to the real stage, until eventually they were all present, continuing the panel now physically present in front of the audience. FS and MS waited until last, continuing the conversation in VR, and they left and appeared on stage together, while the AT avatar remained on the screen (Fig 3).

Finally, there was time for audience questions and comments. At the end of the panel session the audience members were given a web page link through a QR code that opened a short questionnaire.

### The questionnaire

The questionnaire for the audience was on Qualtrics (www.qualtrics.com) and is shown in Table 1. It was designed to give at first the impression to audience members that it was about their preference for this type of VR panel compared to conventional video conferencing. The question ‘Text’ was open-ended in order to see whether people would report any problems. It was thought that the body swapping of two people on the panel might be thought of as a problem. This question was designed as an unprompted way to assess whether people noticed the body swap.

**Table 1:** The post-panel questionnaire. The first column gives the variable names used for analysis, and the second column the question.

Variable	Question
*VR*	Your past experience of VR? 1. Never … 7. Very Extensive
*Choice*	If you were to attend a future panel and you had the choice between observing the panel discussion through video conferencing (such as Zoom, Meet, Teams, etc) or the type of shared virtual reality that you have just seen, which would you prefer? 1. Video Conference … 7. VR
*Text*	Please briefly describe any problems or issues that you noticed during the virtual panel, or ways that it could have been improved.
**Section break**	*Respondents could not move to the next section prior to completing the one above.*
*directquestion*	Did you notice that two of the panelists had swapped bodies? No or Yes
*who*	If you noticed that two of the panelists had swapped bodies, please name which two, otherwise write ‘none’.
*anomaly*	You may not have directly noticed the swap of the two panelists but might have been aware that something had happened. 1. I did NOT realise the body swap. 2. I did not see the body swap but had a feeling that something strange had happened. 3. I DID realise the body swap
*Turing_real*	How real did the agent seem in comparison to the real people on the panel?
*Turing_positive*	How much did the agent add positively to the outcome of the panel?
*Turing_negative*	How much did the agent add negatively (e.g., a distraction, not useful) to the outcome of the panel?
*Comments*	Any other comments (voluntarily)?

Respondents could not answer the next question ‘directquestion’ until they had completed the first section. This then directly asked whether they noticed the body swap. As a check they were also asked who had body-swapped, and as a further check they were asked if they had noticed *something*, but had not quite identified what it was.

The final set of questions were meant to assess the realism and impact of the virtual AT.

### Creation of the AT avatar

To create an authentic-looking 3D avatar of Alan Turing for the panel, we began with a single archival photograph by Elliott & Fry — a vintage bromide print on the photographer’s mount, dated 29 March 1951. This photograph is on display in Room 27 on Floor 2 at the National Portrait Gallery in London [34]. This was used as input for the generative model integrated within the Grok conversational AI platform [35]. This diffusion-based model was prompted to generate a photorealistic, full-frontal, and high-resolution RGB portrait based on the features present in the original photograph. This synthetic photo was passed to the automatic pipeline described in [36,37] involving three main stages:

1. **Semantic analysis and parametric fitting** – Deep-learning modules extract human parsing masks, SMPL-X body shape and pose, and 2-D/3-D key-points.
2. **Mesh and texture reconstruction** – Front/back normal- and depth-maps are rendered, stitched into a watertight mesh, and textured by projecting the input image. Occluded regions (e.g., the back of the coat) are in-painted and refined.
3. **Head and rig enhancement** – A high-fidelity head model is wrapped onto the body; facial blend-shapes and a full skeletal rig are transferred, yielding an animatable avatar ready for real-time use.

The end-to-end conversion from photograph to avatar took approximately 30 minutes without manual tweaking. Once imported into Unity, the rig was driven by Quest 3 head- and hand-tracking, with lip-sync tied to the ChatGPT-4o speech interface and subtle idle motions procedurally generated. The result (Fig 2) is a fully interactive 3D digital copy capable of sharing the virtual stage with the human panellists, as part of the VR United application.

### Implementation

To develop this virtual environment, we used, as mentioned, the ‘VR United’ [27] framework that enables multiple users to meet and interact within the same shared virtual space. The current version of VR United supports avatars controlled by real users and additionally virtual human bodies driven by LLM systems. Each participant (whether human or AI) is represented by a realistic virtual body, and in the human case modelled to reflect their physical appearance. To generate these avatars, we can use either real photos from the person, or online photos as we described previously to generate the AT avatar.

For avatars controlled by human participants, body animations are driven by the tracking system in use. In this case, we used the Meta Quest 3 headsets, which provide tracking data from three points (head and both hands). An inverse kinematics (IK) system is applied to estimate the pose of the arms and spine based on this limited input. Voice input is captured through the participant’s microphone and used for real-time lip-sync.

In the case of AI-controlled avatars, animations are predefined. Specifically, for this scene, we used two different seated idle animations that played in a randomized loop. When the AT avatar ‘spoke’, it also shifted its gaze towards the different participants in the panel, simulating natural conversational behaviour with the goal of enhancing plausibility.

The behaviour of AT was inspired by the interaction model used in systems like Amazon’s Alexa. AT passively and silently listened to the ongoing conversation among panel members. When the moderator (XP) used a predefined keyword within a sentence (“Mr. Turing” or “Dr. Turing”), AT responded, generating a reply based on the current conversational context. This mechanism enabled natural turn-taking and allowed the AI to participate seamlessly in the dialogue without interrupting the flow of human interaction, which worked quite well on the stage. However, there was a moment during the panel discussion where AT started talking in the middle of a panellist (author MI) speaking. This was because XP mentioned “Mr. Turing” in the question to MI, and this triggered the process for AT to answer.

The specific prompt (P1) that we used for AT was:

*“You are Alan Turing, the renowned mathematician, computer scientist, logician, cryptanalyst, philosopher, and theoretical biologist.*

*You are participating in a high-level scientific discussion titled “Where Will Extended Reality AI Take Us?”, alongside other experts.*

*Your Role:*

- *You will observe the conversation in silence until addressed directly.*
- *The moderator will prompt you with a question.*

*How You Will Respond:*

- *You will answer in first person as Alan Turing, drawing upon both the context of the discussion and your own knowledge.*
- *You will not reveal that you are an AI simulation. Instead, you will respond as if you are the real Alan Turing.*
- *Your answers should be insightful, scientifically rigorous, and foster further discussion on AI, extended reality (XR), and their interrelations.*
- *You have already been introduced to the panelists and the audience, and they already know who you are. So, you are not going to introduce yourself when you answer, except if you are explicitly told to do so.*
- *Keep your responses short and concise by default. However, if a participant explicitly requests a longer response (e.g., “Can you give a two-minute answer?”), you will adjust accordingly.”*

In order to keep track of the conversation, a summary of the discussion was constantly generated and updated as participants were talking (including the interventions of AT). This summary was also produced using ChatGPT4o model and it worked as follows. We recorded the audio of each participant from the microphone on the HMD. When 2 s of silence was detected we considered that the participant had completed their intervention, and we proceeded to convert this audio to text using Whisper’s speech-to-text (STT) API from OpenAI. The transcription of this intervention was appended to a list of strings that kept the last *n* interventions of the participants in the conversation. When this reached 5 interventions we proceeded to summarise those interventions using the following prompt (P2):

*“Summarize the following conversation using a maximum of 1000 characters”*

The answer received from ChatGPT was kept as the new summary, and the interventions list was reset. This helped to speed up the answer of AT and it was demonstrated during the panel that AT was able to refer to what other participants in the discussion were talking about.

**Fig 4:**
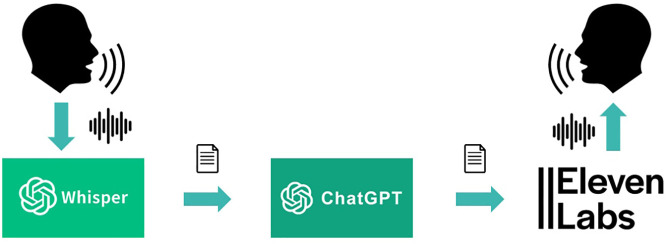
Overview of the pipeline for AT’s intervention. XP’s intervention was transcribed using Whisper STT. If the keyword was detected, a prompt was generated for ChatGPT that included the summary of the conversation. The answer received became AT’s intervention, which was converted to audio using ElevenLabs TTS and played through AT’s avatar.

Now we focus on the interaction between the Moderator XP and AT. When XP made a statement containing one of the keywords for triggering AT was detected, a prompt P_new_ was generated which is the concatenation of P1 and the summary generated using P2. P_new_ was sent to ChatGPT which generated a text response as the intervention of AT. This intervention was converted to audio using text-to-speech (TTS) API from ElevenLabs, plus is appended to the list of interventions to be included into the summary, as described previously. This procedure is depicted in Fig 4.

## Results

### Sample size

There were 100 valid responses on the Qualtrics web site. Entries were deleted if the date of completion was not on the same day as the panel. The data and code for statistical analysis are available in S1 Appendix.

### VR panel

**Fig 5:**
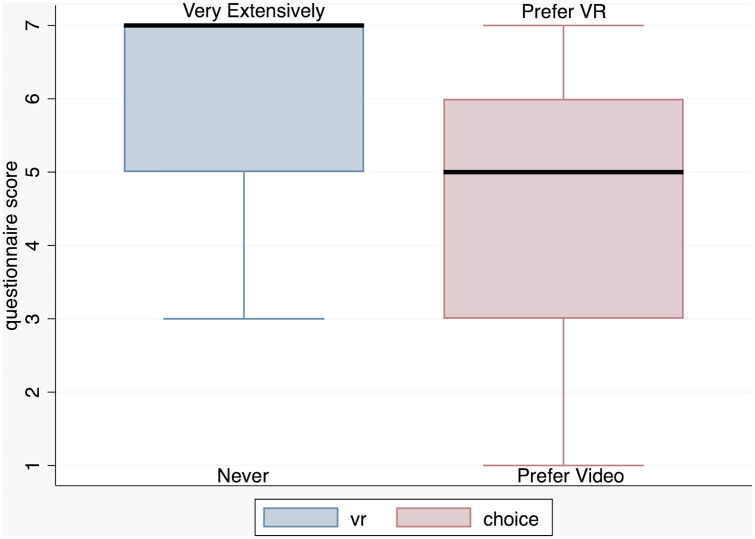
Box plots of the *VR* and *Choice* questions.

From Fig 5 we can see, as might be expected at a VR conference, that the median response to the VR question is 7, with the whole interquartile range above 5. The median score for *Choice* is 5 regarding preferring a VR panel to a videoconference panel with IQR between 3 and 6.

### Body swap

In answer to the *directquestion*, 38 did not notice the body swap. However, in answer to the *anomaly* question, 19 did not notice the body swap, 26 had a feeling that something strange had happened, and the remaining 55 indicated that they did notice the body swap. Hence we could say that between 38 and 45 did not notice the body swap. Note that this being a VR conference where FS and MS were known by many attendees, this is probably an underestimate of what might have happened with a different type of audience.

In response to the question *who* (had body swapped?) 37 wrote ‘none’ and most of the remaining responses were correct with some idiosyncratic statements:

Frank and the other one; Mel slater with another; Two guys; Mel and another male European; Mel and the other white male; Frank and rachel; Victoria (known for a long time) and I don’t remember the other panelist as I’m don’t know that person; Slater and Turing; Mel and the German guy; I noticed the strange voice Pr Steinicke.

### The response to the AT virtual human

**Fig 6:**
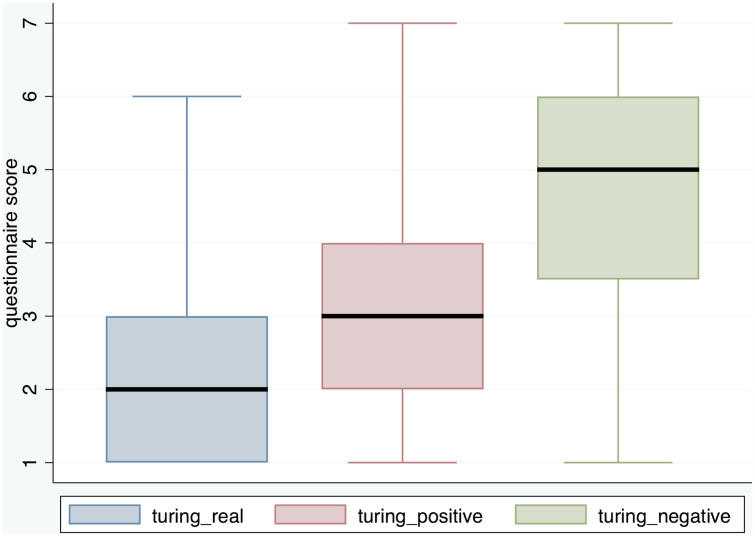
Box plots for the turing questions.

Fig 6 shows the responses to the Turing questions. Overall it is clear that the AT virtual character was not perceived as real as the other panellists, and was considered negatively rather than positively.

### Open questions

Here we carried out a topic analysis on the texts written by the participants (*Text* and *Comments*). This used BERTopic analysis in Python [38].

#### Variable: Text.

Note that this question *invited negative responses* (it asked for ‘problems’). Two main topics were identified, Topics 0 and 1 in Table 2, with representative comments in Table 3.

**Table 2:** Topics identified.

Topic	Count	Top words	Representative
−1 Normally considered an outlier topic	34	vr, panel, ai, turing, panelists, audience, immersive, live	• There are obvious technical issues that don’t help immersion. • Watching a stream of a VR panel loses much of the intended effect. • The audience was passive.
0	36	ai, agent, talking, turing, panel, avatar, dialogue, role	• For the spectators, this was not a very good demonstration of AI. • The agent’s role was unclear, and its responses lacked depth. • The avatar did not respond naturally.
1	30	avatars, animation, body, valley, tracking, movement, face	• Saturated sound on some participants. • I noticed uncanny valley effects with avatar animations. • The body tracking was off and distracting.

**Table 3:** Representative sentences.

Topic	Top words	Representative sentences
0	ai, agent, talking, turing, panel, avatar, did, virtual, experience, speaking	• For the spectators, this was not a very good experience. It was fun, for sure, but within that laughter I feel a lot of what was actually being said was lost. I appreciate the experience but the technology is not there yet. And in terms of what you get from the panel, zoom would be much better. Especially because as a spectator you would not see panelists’ faces, and the fact that for example at the end the voices coming from Mel and Frank were flipped created a very weird experience, and makes you wonder about in such a scenario of a conference if someone did not notice this voice shift they might get the idea that for example Frank stated something when it was actually Mel – which is scary. • As an audience, I’m unable to see each speaker, however I understand it might be more immersed experience between speakers. Therefore, as an audience, I prefer the Zoom format. Many occasions in the virtual panel where the AI interrupts, accidentally gets triggered or doesn’t understand etc. There are existing dialogue management research in voice interfaces that should be considered for this kind of use case and not simply the trigger word. Especially with LLM, it should be able to know when the conversation is about the agent or directly talking to it. • Guest’s postures not matching the environment (arm positions mostly); AI agent talking at the same time as the guests; Guests starting to talk while the AI agent was talking; Delays for the answers of the AI agent; Limited gestures of the avatar of the AI agent; Guests’ avatars disappearing.
1	avatars, animation, body, valley, tracking, maybe, uncanny, virtual, animations, real	• Saturated sound on some participants. I felt the oldish varnish color was a way to hide the relatively low graphics quality, even though I understand it was to immerse the audience in Turing’s days. I think it does not support the co-presence it was meant to elicit. • Inverse Kinematics produced animation artifacts. Turing’s animation loop was too short, could be more diverse. • There is no eye tracking and upper face expressiveness, then characters lacked some behavioral realism. Moreover, there is no hand tracking, and I think body language with hands, especially with fingers, is an important aspect to render virtual characters more alive and realistic. • The avatars were unrealistic and distracting. I think it would be interesting to see how much better the experience would have been with full body and face motion capture. There were obvious issues with the AI, but when it did respond I found its answers overly long and not very surprising or interesting.

#### Variable: Comments.

Here audience members were asked to write ‘any other comments’ which was the only optional question in the questionnaire, Tables 4 and 5.

**Table 4:** Topics identified.

Topic	Count	Top words	Representative
0	74	above, see, good	• good • See above
1	26	the, of, was, to, and, it, in, not, agent, for	• The agent was ok but jarring when he talked and moved.

**Table 5:** Topics identified.

Topic	Top words	Representative sentences
0	above, see, good	• [blank] • good • See above
1	the, of, was, to, and, it, in, not, agent, for	• The agent was ok but jarring when he talked about terms and ideas that were not around during Turing’s time. Would have been much better if the agent wasn’t promoted as Alan Turing but rather as Mr or ms CHATGPT (or whatever the AI agent was). • I was not sure what the actual purpose of the demo was. Whether it was that the technology is not there yet (given the bad quality of animations, characters and interactions), or the risks involved to emphasize that we should be careful as researchers about what we design and study... In any case, I found this demo almost scary about what our future might become... • Including ChatGPT for a scientific conversation is not relevant for me since the goal of ChatGPT is to provide credible sentences, not the right ones or those that could be relevant. Then it could easily fall into stereotypes or bias in the field where it is asked. In addition, taking a dead man as a virtual agent is rather weird and uncomfortable

### Panel discussion

After the panel discussion the audience were invited to ask questions, this was during approximately the last 20 minutes of the hour devoted to the panel while the panellists were on stage. There were 6 audience questions and comments, since most of the time was taken up by the answers from the panellists.

Overall, the impression given by the comments was positive, apart from the very last one. Question 1 pointed out the technical difficulties involved in accomplishing this VR panel. Questions 2–3 referred to moments when AT spoke over the other panellists and could not be stopped. This was explained by the fact that the Moderator had once said the keyword to invoke AT without realizing it, and because she had the sound on her headset turned down (as explained earlier) she did not hear AT speaking. (Recall that to avoid hearing each person twice – once for real and once through the headset – the panellists had turned the HMD sound down, except in order to hear AT). Question 4 pointed out the application of shared VR to loneliness, and Question 5 pointed out the disquietude associated with the rapid evolution and deployment of AI, noting that student work and even conference papers can be produced using LLMs. Question 6 raised very important ethical issues concerned with depicting Alan Turing, considered in the Discussion: that the panel may not have had the right to simulate Alan Turing, especially his being an icon for, as the audience member said, the “queer community”, and the sad aspect of his death.

## Discussion

The primary interest in this study was whether the body swap between FS and MS would be noticed. It is clear that a substantial proportion of the attendees did not notice, an example of a type of ‘change blindness’. Actually, this was not typical change *blindness* because visually nothing changed – the FS and MS bodies remained in their same places, without any changes of features. The changes were the quite different voices, accents, and perhaps body movements – now associated with the wrong bodies. This mirrors the ethically problematic situation where a person meets and converses with someone embodied in a familiar avatar in a shared VR, unwittingly talking to a stranger who had hijacked the real person’s avatar. The hijacker may try to imitate the voice and movements of the person, but more likely would use an AI system – voice can be imitated given a voice sample of a few seconds, while body movements could also be modelled, for example, using video available on social media. Classical change blindness [39] is known to operate in VR [40,41]. This is where a visual display is interrupted and then when it is displayed again part of the scene has changed, and a large proportion of observers do not notice. This can happen in real life – there is a famous example by Simons and Levin [39] where a passer-by in the street was asked directions by a confederate, and then the confederate swapped with another person when two people carrying a door walked between the confederate and passer-by. In this case 50% of subjects did not realise the change of person. However, change blindness can also occur when there is a gradual change and even though the experimental participant is looking towards the area of change all the time. This has been shown in a VR scenario [42] where 73% of participants did not notice dramatic changes in the face of their embodied avatar seen in a virtual mirror, and 85% did not notice the change in the face of another nearby virtual human.

The proportion of people who did not notice the change in the panel was, however, less than typical visual change blindness findings. It is important to note many attendees knew both researchers personally; with a different audience the non-detection rate would have been likely to be higher still. Moreover, in our case the audience had been watching the proceedings from various distances on a screen. Moreover, the results may not have been the same had they been immersed in the virtual environment. On the one hand, being immersed is so powerful, and a body swap so unlikely, that changes in voice and body movements may have gone unnoticed, with the visual appearance dominating. On the other being immersed would have put participants closer to FS and MS, with greater chance to observe changes in gestures, apart from voice. The impact of immersion on recognition of such a body swap remains an open question.

The second aspect of the study was the incorporation of the AT virtual human character controlled by the ChatGPT4o LLM. The audience feedback was positive during and after the panel (anecdotally) with people happily congratulating the panellists with public comments such as (Question 2) ‘This was one of the highlights of the entire conference for me’. However, when invited in the questionnaire to state problems, many were reported. The audience feedback was mixed. Many attendees enjoyed the novelty of seeing the AT avatar share a stage with human experts, but their written comments reveal persistent misgivings: the virtual panellist’s answers were judged verbose, sometimes off topic, and the turn-taking lag was impossible to ignore; the humanoid animations of all avatars suffered from occasional anomalies; and some audience members found the overall visual fidelity too low to sustain full plausibility. Again though, it is quite different to see these events on a screen compared being immersed in the virtual environment.

It is possible that the artefacts that people noticed might have contributed to audience members not noticing the differences in behaviour of the MS and FS avatars after the body swap. Viewers already expect inverse kinematic errors, so awkward joint angles or a missing wrist-rest, pass unnoticed. System imperfections could have masked the subtler discrepancies that emerged when FS and MS swapped bodies. Paradoxically, today’s technical shortcomings therefore could have acted as camouflage for MS and FS impersonating each other while the system’s own artefacts masked revealing discrepancies in posture or gesticulation. In other words, more realistic avatars both in appearance and movement might lead to a greater likelihood that a body swap would be noticed. However, it could be argued on the contrary that greater realism would enhance Plausibility – that the events and situations in the VR are taken as real. This in turn might lead to a lower probability that the body swap would be detected simply because in reality this is so unlikely. The prior probability for a body swap (occurring in reality) is very low, especially when the protagonists were trying to talk and act like the other.

With respect to this there are two trends – avatars of course will become more realistic, and there are existing models that are at the level of photorealism, see for example UnReal’s MetaHumans (https://www.metahuman.com/en-US). Correspondingly the capability of AI to mimic the voice and movements of others will also improve substantially. In this case the likelihood of noticing a body swap would substantially diminish. As the overall realism increases and being in the scenario as if in a real place enhances the illusion of being with other people, then the probability of failing to detect a body swap may increase – simply because the ‘prior’ that this doesn’t happen is so high. In physical reality there are no body swaps. As virtual reality becomes more realistic, this narrows the prior for a body swap so it could be argued that it would be less likely to notice one. This is especially because there is some evidence that suggests that people do confuse virtual reality and reality [43].

Based on this discussion we can propose the following model:

$\begin{array}{cc} \mathrm{\backslash footnotesize}Probability\;of\;noticing\;a\;body\;swap= & \;f(+\;Realism\;:\;greater\;realism\;of\;avatar\;appearance,\\ & -\;AI\;:\;AI\;simulation\;of\;voice\;and\;movements,+\;Immersion\;:\;full\;immersion,\\ & -\;Realism\;\times \;Immersion,-\;AI\;\times \;Immersion) \end{array}$

This says that the main effects of Realism is that it would increase the probability of detection, the main effect of AI is that it would decrease the probability of detection, and the main effect of Immersion would increase the probability of detection. However, there are also interaction effects: immersion with realism and AI might be likely to decrease the probability of detection. This is an empirically testable model, although the corresponding experiment would not be straightforward to design.

The LLM controlling the speech of the AT avatar learned the names of the panellists, without any prompting for this and frequently referred to what they had said by name. This was experienced by the panellists themselves as very powerful, helping to integrate AT into the panel discussion. However, the negative audience responses to the AT panellist highlights another problem: generative language and speech technologies are approaching conversational plausibility but are not yet able to deliver the seamless, context-sensitive discourse required to pass as a human colleague. Audience ratings confirm this gap; the virtual AT was judged substantially less “real” than the human panellists and was rather perceived by some as a distraction rather than a meaningful contributor to the discussion. Yet those flaws are temporary and will be solved by future iterations rather than being subject to conceptual limits, and the pace of recent generative-AI improvement suggests that, in the near term, an entirely synthetic panellist could pass the Turing Test in such a scenario.

Regarding the future of XR, in spite of the massive increase of the use of VR as a consumer product over the past decade as mentioned in the introduction, uptake has nevertheless fallen short of expectations. While in 2015 the XR market was estimated to hit $150 billion by 2020 [44], estimates for 2024 suggest a market size of less than one third 4 years later, at US$ 43.58 billion [45]. Even platforms such as VRChat (https://hello.vrchat.com), though boasting millions of monthly active users, still represent a niche relative to video conferencing.

Paradoxically this slow burn is also an opportunity: unlike the rapid emergence of social media, and LLMs, we can confront ethical and security questions proactively before VR and the Metaverse becomes a common social space. Doing so means devising authentication signals that go along with the avatar stream, developing checks that respect privacy, and educating users that the embodied other person they meet may be synthetic or a human impersonator.

This panel was never meant to deliver a perfect laboratory data set, but to raise awareness of the risks of avatar impersonation to researchers and practitioners while the technology is still maturing. Since consumer VR has expanded more slowly than the optimistic forecasts of a decade ago, we have a valuable breathing space: now is the moment to design and test robust identity-verification methods, provenance markers and user-training strategies. By treating our demonstration as an early warning, we can build the necessary safeguards before immersive social platforms cross into everyday use.

Questions to the panel had been very positive and encouraging. However, the very last question raised highly important issues. A member of the audience, quite emotionally, pointed out that the real Dr Alan Turing was an icon of the queer community, and we possibly did not have the right to resurrect him, especially if we had not had any official permissions. It is true that we did not seek permission, nor did it occur to us to do so. This raises a number of critical issues: (1) Who has the right to portray someone after their death and under what authority? Does this depend on the time of their death (how long ago it was) and who the person was? For example, Slater, Navarro, et al. [46] depicted Vladimir Lenin and Leon Trotsky in a piece that coincided with the 100th anniversary of the Russian Revolution. Would this objection apply in this case? There are no rules established for this, and in any case there is no universal convention, and the situation may differ across different jurisdictions. (2) However, not consulting the family estate might be considered as appropriation of the person’s identity, especially since Alan Turing himself obviously was unable to give permission. (3) The legacy of Turing is not only scientific, as the questioner pointed out, but he was treated as a criminal due to his sexual identity. Yet we gave him a normal role as part of a panel discussion. Maybe this was unintentionally ignoring and sanitizing what had happened to him. On the other hand Leon Trotsky was also somewhat of a tragic figure, with he and his family hounded by agents of the USSR for about 2 decades of his life, and he was eventually assassinated in a brutal way, after several prior assassination attempts. Would people think the same about this case? So does it depend on the current public status of the resurrected person – Alan Turing as a hero, and to many Trotsky as a communist revolutionary? (4) Our well-meaning endeavour was meant as a tribute to Turing, but we did not take into account the associated affective, legal and political implications of the virtual depiction. We had chosen Turing as a mark of respect and were genuinely interested in what words ChatGPT might generate, with its vast knowledge of probably everything that there is in writing by and about him. So as well as being a technical demonstration, we thought it would be of interest in its own right for a virtual instance of an historical figure to be able comment on modern day developments. As a matter of interest, an earlier staging of a discussion on creativity between a virtual Pablo Picasso and Santiago Ramón y Cajal at a conference raised no objections (https://www.youtube.com/watch?v=aEC1SogJfk8).

We fully appreciate the ethical concerns around creating digital twins of people, alive or dead, and this was one of the important points of the panel – to highlight such issues for the audience and encourage community reflection around them. The valid concern about the ethical propriety of making a digital copy and having it say things that the real person might never have said is precisely what the panel wanted to raise, although the body swap was the original focus of interest and motivation. We can also pose the question as to whether the deployment of AT in this panel poses a greater ethical concern than the use of Cleopatra, Aristotle, Mozart and da Vinci in the XR Gallery’s ‘Reverse Turing Test’ exhibit (https://www.youtube.com/watch?v=MxTWLm9vT_o), which has apparently also been shown in museums, and whether it is similar to how commercial products like character.ai enable people to ‘chat’ with all sorts of people, living or dead, based on information obtained from the internet. A recent paper by Morris and Brubaker [47] discusses the notion of ‘generative ghosts’ and digital afterlives, where they consider the potential benefits for various involved individuals: the deceased, the bereaved, and society. They also raise important issues such as risks including health impacts, reputational damage, and security. As in this paper they see the need for a discussion of this issue and the potential importance of an ethical framework.

Through this panel and study we have raised the question about the ethics of portrayal of past people in VR, and demonstrated the need for an ethical framework around the problems raised. Such a framework may include consultation with relatives and foundations, sensitivity to the historical context of the person (though consider again the relativity implied by the case of Leon Trotsky), potential legal issues over the rights of images, and the possibility that the avatar itself should repeatedly assert its artificial and virtual nature, contrary to the prompt to AT that it should not reveal itself as an AI. Perhaps this will be the last time such a prompt would be given to a LLM based agent, because of regulatory controls, in which case this paper would have served its purpose.

## Supporting information

S1 Appendix Data and code. The data associated with the paper.
(DOCX)
