# Supplementary material for: Where extended reality and AI may take us: Ethical issues of impersonation and AI fakes in social virtual reality
Source: PLoS One. 2026 Apr 2;21(4):e0340829. doi: 10.1371/journal.pone.0340829 (PMC13046158; doi:10.1371/journal.pone.0340829)
Supplement: S1 Appendix — The data associated with the paper. (DOCX) [file pone.0340829.s001.docx]

S1 Appendix

Data and Code

The data associated with the paper can be found at:

<https://www.kaggle.com/datasets/melslater/ieee-vr-2025-vr-panel-data>

The code for statistical analysis can be found at:

<https://www.kaggle.com/code/melslater/ieee-vr-data-programs>

In order to actually run the analysis login to Kaggle (create a login if you do not have one) and then select ‘Edit’ and you can execute each block of code by selecting the small arrow to its left.

The results may not be identical to those published in the paper because of the stochastic nature of the methods used.
